# Prevalence of undernutrition and associated factors among children aged 6–23 months: a cross-sectional analysis from South-East Ethiopia

**DOI:** 10.1017/jns.2023.109

**Published:** 2023-12-27

**Authors:** Gosa Girma Ararsa, Meheret Tena Getachew, Tona Zema Diddana, Fikadu Reta Alemayehu

**Affiliations:** 1 School of Nutrition, Food Science and Technology, College of Agriculture, Hawassa University, P.O. Box 05, Hawassa, Ethiopia; 2 Ethiopia Civil Society Coalition for Scaling Up Nutrition (ECSC-SUN), Scaling Up Nutrition (SUN), Civil Society Network, P.O. Box 384, Ethiopia Country Office, Addis Ababa, Ethiopia

**Keywords:** Lemubilbilo, South-east Ethiopia, Undernutrition, 6–23 months aged children, ANC, antenatal care, AOR, adjusted odds ratio, ARI, acute respiratory illness, ASF, animal source food, BF, breastfeeding, CF, complementary feeding, COR, crude odds ratio, EDHS, Ethiopian Demographic and Health Survey, DDS, dietary diversity score, GMP, growth monitoring and promotion, HAZ, height/length-for-age Z-score, HIFAS, household food insecurity access score, IYCFP, infant and young child feeding practices, MAD, Minimum acceptable diet, mm, millimeter, OFSP, orange fleshed sweet potato, ORS, oral rehydration solution, PNC, postnatal care, WAZ, weight-for-age Z-score, WHO, World Health Organisation, WHZ, weight-for-height/length Z-score

## Abstract

To meet the 2030 goal to end all types of malnutrition, thoroughly investigating and addressing context-specific factors of undernutrition is crucial. Therefore, this study assessed the prevalence of undernutrition and associated factors among children aged 6–23 months in South-East Ethiopia. A community-based cross-sectional study was conducted on 580 randomly sampled mother–child pairs in February 2022. Socio-demographic, dietary intake, household food security (HFS), maternal knowledge and practices of child feeding, and the child's weight and height data were collected. A multivariable logistic regression analysis was done. The prevalence of stunted, wasted, and underweight children was 32⋅1, 7, and 9 %, respectively. Being male (AOR = 1⋅75), not using the growth monitoring and promotion (GMP) service (AOR = 1⋅50), household food insecurity (HFI) (AOR = 1⋅67), lack of improved water (AOR = 2⋅26), and bottle-feeding (AOR = 1⋅54) were significantly associated with stunting. Being male (AOR = 3⋅02), having low maternal knowledge on child-feeding practices (AOR = 3⋅89), not listening to the radio/television (AOR = 3⋅69), having a history of fever (AOR = 3⋅39), bottle-feeding (AOR = 3⋅58), and HFI (AOR = 3⋅77) were significantly predicted wasting. Being male (AOR = 3⋅44), not using GMP service (AOR = 2⋅00), having a history of fever (AOR = 4⋅24), lack of knowledge on optimal breastfeeding duration (AOR = 3⋅58), low maternal knowledge on child feeding (AOR = 2⋅21), HFI (AOR = 2⋅04), and lack of improved water (AOR = 3⋅00) showed significant association with underweight. In conclusion, stunting is alarmingly common while wasting and underweight are sub-optimal. Prevention of infectious disease, providing basic education for fathers, ensuring HFS; enhancing media access, maternal knowledge about IYCFP and improving water access; and GMP service utilisation are crucial to improve child nutrition.

## Background

Undernutrition poses a major threat to a child's survival, physical growth, cognitive development, future productivity,^(1,2)^ and school performance.^(3,4)^ Children who suffer from early stunting, particularly in the first 1000 days, may never reach their full physical and intellectual potential for the rest of their lives.^(1)^ Globally, child undernutrition continues to be a public health concern. In 2020, globally, 149⋅2 million under-five children were stunted, 45⋅4 million were wasted,^(5)^ and about 45 % of all deaths attributed were to undernutrition.^(6)^ In 2019, 57⋅5 million (29⋅1 %) children were stunted in Africa.^(7)^ and Sub-Saharan Africa (SSA) is the world's leading region where a reduction in the number of stunted children is not in sufficient rate to meet the global target. Between 2012 and 2019, the prevalence of stunting in SSA decreased only by 3⋅4 % (from 34⋅1 to 31⋅1 %).^(8)^

Ethiopia is one of the SSA countries facing child undernutrition problems. In the last 10 years, nationally, stunting showed only a 7⋅6 % decrease (from 44⋅4 % in 2011 to 36⋅8 % in 2019). Underweight is reduced only by 12 % (from 28⋅7 % in 2011 to 21 % in 2019), and acute malnutrition is fluctuating between 7 and 9⋅7 %.^(9,10)^ The cost associated with child undernutrition is also very high in Ethiopia. For instance, annually, Ethiopia loses about 16⋅5 % of the gross domestic product (GDP) due to undernutrition.^(11)^ About 28 % of under-five child death is attributed to undernutrition in Ethiopia.^(12)^ Thus, investing in factors associated with child undernutrition is critical since the economic returns from investment are high and it saves a lot of lives.^(1)^

Despite many intervention approaches such as national nutrition programme-II undertaken, Ethiopia is not on track to achieve the 2030 commitment to end all forms of malnutrition, and the 2015 Seqota declaration target set for zero stunting among children of under 2 years by 2030.^(13)^ Currently, the national food and nutrition strategy has implemented with special emphasis on the first 1000 days. Furthermore, the maternal and child health and nutrition services such as antenatal care (ANC) and postnatal care (PNC); child vaccination, vitamin A supplementation, and nutrition counselling are ongoing in the Woreda to improve child nutritional status. However, there is paucity of data on the magnitude of undernutrition and its drivers in the current study area. Unless thoroughly investigating and addressing multiple context-specific factors including the effectiveness of interventions, these goals can't be achieved. The finding of this study could be used to provide essential information to concerned bodies to compel fast action specific to local contexts and give some guidance in intervention areas. Hence, this study assessed the prevalence of undernutrition and associated factors among children aged 6–23 months in South-East Ethiopia, 2022.

## Materials and methods

### Description of the study area

This study was conducted in Lemubilbilo Woreda of Oromia Region, South-East Ethiopia. It is located at about 235 km to the South-East of Addis Ababa. The area has an annual rainfall of 1100 mm, of which over 85 % is during the main rainy season (June to November). According to the Woreda Agricultural Office, the altitude of the area is 2567 meters above sea level and the average annual temperature ranges from 6 to 26 °C. The major crops grown in the area are wheat, barley, maize, millet, teff, and beans.^(14)^ Based on the Lemubilbilo Woreda health office report, the total number of mothers/caregivers having 6–23 months aged children during the data collection period was 3432. Woreda had 32 Kebeles (the lowest administrative unit) with a total population of 226 780. There were seven public health centres and 27 health posts in charge of the whole population of Woreda during the study period.

### Study design and study period

A community-based cross-sectional study was conducted in February 2022.

#### Source populations and study populations

Mothers/caregivers with their children aged 6–23 months who live in Lemubilbilo Woreda were source populations. A randomly selected mother–child pairs from the source population were study populations. The study unit was mother/caregiver having children aged 6–23 months, lived in the randomly selected Kebeles and participated in the actual data collection interview.

#### Sample size determination

The prevalence of stunting (37 %) from previous study report by Ethiopian Public Health Institute (EPHI) was used to determine sample size.^(10)^ A 95 % confidence interval, critical value (1⋅96) and margin of error (0⋅05) was taken. Assuming the heterogentiy among clusters (Kebeles), the design effect (DE) of 1⋅5. The DE from previous national survey was 1⋅18 for stunting, 1⋅04 for underweight, and 1⋅68 for wasting in the region where this study was conducted.^(10)^ Hence, this study used 1⋅5 which is closer to average of nationally reported DE for specific undernutrition. For non-response rate, 10 % of the calculated sample size was added. A total of 591 mother–child pairs were determined for the interview. Because of exclusion and withdrawal of mothers during interview, data were collected from 580 mother–child pairs.

#### Sampling techniques and procedure

Two-stage sampling technique was followed. First, 14 Kebeles (the smallest administrative units) were selected from a total of 32 Kebeles in the Woreda by lottery method. Then, a list of households having children aged 6–23 months in the selected Kebeles was made as a sampling frame. For mother having twins, one child was randomly selected and included into sampling frame. Then, study participants were recruited by a simple random sampling technique.

### Inclusion and exclusion criteria

All volunteered mothers/caregivers who had 6–23 months aged children and lived for at least 6 months in the study Woreda before the data collection period were included. Caregivers who could not respond because of health problems and severely sick children were excluded.

### Data collection instruments and procedures

The Ethiopian demographic and health survey (EDHS) 2016 questionnaire was adapted to collect socio-demographic and household wealth status indicators (ownership of durable assets, domestic animals, household facilities, and productive assets).^(9)^

Household food security status was assessed by using Family Health International and Food and Nutrition Technical Assistance (FHI 360/FANTA) indicator guide for household food access. Mothers/caregivers were interviewed for nine occurrence questions, followed by each frequency-of-occurrence for the specific experience of food insecurity 4 weeks before the data collection date. The indicator categorises households into food secure, mildly food insecure, moderately food insecure, and severely food insecure.^(15)^

Selected infant and young child feeding practice (IYCFP) indicators were adapted from WHO/UNICEF (2021).^(16)^ Minimum meal frequency (MMF) and minimum child dietary diversity score (MDDS) were assessed by 24 h recall method. Minimum acceptable diet (MAD) was computed from MDDS and MMF. The knowledge of mothers/caregivers about the recommended child feeding practices: early initiation of breastfeeding, breastfeeding frequency, exclusive breastfeeding, benefits of breast milk feeding to child, optimal duration of breastfeeding, benefits of starting complementary feeding at 6 months, age appropriate type food and its preparation, responsive feeding, risk of bottle-feeding, minimum acceptable diet, minimum child dietary diversity and critical hand washing times were assessed. A mother scores ‘1’ if she knew the practice or ‘0’ if she didn't know these IYCFPs. Composite indicator of knowledge on appropriate child feeding practices was created. Then, knowledge level was classified as ‘good’ if a mother scored ≥75 % to indicate sufficient knowledge on recommended IYCFP or ‘low’ if a mother scored <75 % to indicate a mother/caregiver has insufficient knowledge.

Obstetric, maternal, and child health service utilisation, and child illness history were assessed. In addition, illness history for infectious diseases like acute respiratory illness, diarrhoea, fever; and growth monitoring and promotion (GMP) service utilisation data were collected.

The weight of child measured by standard a weighing scale reading to the nearest 0⋅1 kg. Height/length was measured to the nearest 0⋅1 cm using a standardised measuring board. The height/length of the children was measured while lying on their back on the length board. Data about Date of Birth of children was taken from vaccination cards and health centre registration cards. For those children who had no card, mothers were asked to report the dates of birth. Then, anthropometric indicators: weight-for-age z-score (WAZ), weight-for-height/length z-score (WHZ), and height/length-for-age z-score (HAZ) were computed using the 2006 WHO child growth standard reference value.^(17)^ A *Z*-score value of −2SD is used as a cutoff point to classify a child as well-nourished or undernourished.

### Data quality control

Primarily, the questionnaire that prepared in English was translated into the local language (Afan Oromo). Then, it was back translated into English to check its consistency. The tool was pre-tested by 5 % of the calculated sample size in the Lagana Jabi Kebele which is the village other than the actual data were collected. Reliability was tested by the test re-test method. Cronbach's alpha (*α*) coefficient was determined. The questionnaires with *α* > 0⋅7 were included. The validity of the questionnaire was checked by Pearson's correlation (*r*) for each domain of questions. The questionnaires with a correlation coefficient of >0⋅05 were declared valid. Three days training was given to ten data collectors with a Bachelor of Science (BSc.) degree in human nutrition. To minimise professional bias, data collectors who had no previous contact with respondents were included. Additionally, data collectors were clearly oriented to avoid leading respondents to correct answer, confirming and summarising the respondents feeling. The training was provided by researchers. Training was focused on the overall survey, consent taking process and anthropometric measurement tools. Weight and height were measured twice and an average was used as a final result. Data quality and completeness were checked day to day.

### Data processing and analysis

The data were coded and entered into a statistical package for social science (SPSS) program version 20.^(18)^ Data were checked for missing value. Normality was checked by the Kolmogorov–Smirnov test. The variance inflation factor (VIF) was checked to assess multicollinearity. Household durable assets were dummy-coded (dichotomised) into a nominal level. Wealth index was computed by principal component analysis (PCA). Weight and height/length measurement data were entered into WHO Anthro (2010) version 3.2.2.^(19)^ The outcome variables were dichotomised as stunted/non-stunted, underweight/not underweight and wasted/not wasted. Binary logistic regression analysis was run to assess a significant association of independent variables with undernutrition. Candidate variables with *P*-value less than 0⋅2 in the binary logistic regression were entered into multivariable logistic regression. The forward conditional model was selected in multivariable analysis. At 95 % confidence interval, a variable with *P* < 0⋅05 was declared statistically significant. The strength and direction of association were presented by adjusted odds ratio (AOR).

### Ethical consideration

This study was conducted according to the guidelines laid down in the Declaration of Helsinki and all procedures involving human subjects/patients were approved by the Institutional Review Board of Hawassa University (IRB/015/13). Informed written consent was obtained from all study participants. Information provided by the participants was held confidential.

### Operational definitions

#### Minimum child dietary diversity

Percentage of children 6–23 months of age who consumed foods and beverages from at least five out of eight defined food groups during the previous day.^(16)^

#### Minimum meal frequency

When breastfeeding child aged 6–8 months be provided complementary foods two to three times per day, three to four times with additional nutritious snacks offered one to two times per day for 9–23 months or four to fivemeals per day for non-breastfed children.^(20,21)^

#### Minimum acceptable diet

Percentage of children 6–23 months of age who consumed minimum dietary diversity and received minimum meal frequency during the previous day.^(16)^

Food security: a state in which all people at all times have both physical and economic access to sufficient food to meet their dietary needs for a productive and healthy life.^(22)^

#### Good knowledge of IYCFP

If mother/caregivers correctly answer ≥75 % of recommended infant and young child feeding practices questions, and otherwise classified as low knowledge of IYCFP.

#### Optimal duration of breastfeeding

Feeding breast milk in addition to complementary food for at least 2 years.

#### GMP service utilisation

Child weighed to GMP service at least five and four times for 6–11 and 12–23 months of age per year, respectively.^(23)^

#### Improved water source

In this study, improved water source mean water source that is accessible on premises, available when needed and free from faecal and priority chemical contamination, and includes piped water, boreholes or tubewells, protected dug wells, protected springs, and packaged or delivered water.^(24)^

## Result

### Socio-demographic and economic characteristics

Of the total of 591 mother–child pairs recruited, 580 (98⋅1 %) completed the interview. Based on sex, nearly equal proportion of children (male = 51 % and female = 49 %) were involved. Over 70 % of mothers and fathers attended formal education. More than half (53⋅8 %) of participants had >4 family members in the household. Two-thirds of children (66 %) were 12–23 months aged, and the rest were 6–11 months by age. Nearly 40 % of children were from the poorest and poor households. Over half (53⋅4 %) of the participants were at least mild food-insecure and 46⋅6 % were food-secure. About 60 % of households had no improved water access and 17 % had been using unimproved latrines during study period, respectively (Table 1).

**Table 1.** Socio-demographic and economic characteristics of caregivers mothers/caregivers with children aged 6–23 months in South-East Ethiopia (*n* = 580), 2022

Variables	Frequency	Percent
Sex of the child		
Male	296	51⋅0
Female	284	49⋅0
Maternal education		
No formal education	168	29⋅0
Formal education	412	71⋅0
Paternal education		
No formal education	124	21⋅4
Formal education	456	78⋅6
Occupation of mothers/care givers		
Housewife	508	87⋅6
Others	72	12⋅4
Occupation of father		
Farmer	541	93⋅3
Merchant	31	5⋅3
Others	8	1⋅4
Religion of caregivers		
Orthodox	142	24⋅5
Muslim	390	67⋅2
Protestant	45	7⋅8
Waqefata	3	0⋅5
Current marital status		
Married and live together	564	97⋅2
Others	16	2⋅8
Family size		
≤4 person	268	46⋅2
>4 person	312	53⋅8
Household economic decision maker		
Father (Husband)	356	61⋅4
Both father and mother	185	31⋅9
Mother (wife)	19	6⋅7
Age of mothers at birth		
≤21 years	78	13⋅4
>21 years	502	86⋅6
Age of children		
<1 year	197	34⋅0
1–2 years	383	66⋅0
Number of under two children in the household		
One	556	95⋅9
More than one	24	4⋅1
Wealth quantiles		
Poorest	116	20⋅0
Poor	117	20⋅2
Medium	146	25⋅2
Wealthier	71	12⋅2
Wealthiest	130	22⋅4
Household food security status		
Food secure	270	46⋅6
Mild food insecure	27	4⋅7
Moderately food insecure	143	24⋅7
Severely food insecure	140	24⋅1
Latrine		
Unimproved	100	17⋅2
Improved	480	82⋅8
Household water source		
Unimproved	349	60⋅2
Improved	231	39⋅8

### Obstetrics, child health and service utilisation

The preceding birth interval of nearly one-fifth (22⋅2 %) of mothers was less than or equal to 24 months. Only eight (1⋅4 %) of mothers attended recommended number of ANC services (≥ 4 visits). About 36 % of mothers gave birth at home and 63⋅8 % had no PNC checkup. About 39 % of children did not use age appropriate GMP service. The prevalence of acute respiratory infection (ARI), diarrhoea, and fever 2 weeks before the date of survey was 17⋅8, 36⋅8, and 13⋅8 %, respectively. Only 36⋅8 % of the diarrhoea-diseased children had received ORS (Oral Rehydration Solution) (Table 2).

**Table 2.** Obstetric characteristics, child health and health service utilisation of mothers/caregivers with children aged 6–23 months in South-East Ethiopia (*n* = 580), February 2022.

Variables	Frequency	Percent
Birth order		
First	141	24⋅3
Second	137	23⋅6
Third	128	22⋅1
Fourth and more	174	30⋅0
Preceding birth interval		
No previous birth	141	24⋅3
≤24 months	129	22⋅2
>24 months	310	53⋅4
ANC attendance		
Never attended	59	10⋅2
1 time	91	15⋅7
2 times	237	40⋅9
3 times	185	31⋅9
4 and more times	8	1⋅4
PNC check-up		
Never	370	63⋅8
1–2 days	182	31⋅4
>3 days	28	4⋅8
Place of birth		
Home	212	36⋅6
Health facility	368	63⋅4
Following growth monitoring and promotion		
No	228	39⋅3
Yes	352	60⋅7
ARI in past 2 weeks		
No	447	82⋅2
Yes	103	17⋅8
Diarrhea in past 2 weeks		
No	455	78⋅4
Yes	125	21⋅6
Received ORS during diarrhoea		
No	79	63⋅2
Yes	46	36⋅8
Sick fever in previous 2 weeks		
No	500	86⋅2
Yes	80	13⋅8
Received vitamin A drops yesterday		
No	539	92⋅9
Yes	41	7⋅1

ORS, oral rehydration solution, ARI, acute respiratory illness; PNC, postnatal care; ANC, antenatal care.

### Knowledge of mothers/caregivers on child feeding practices

Overall, 62⋅9 and 37⋅1 % of mothers/caregivers had good and low knowledge of IYCFP, respectively. Of the mothers/caregivers having good knowledge, 190 (52⋅1 %) got IYCFP information from health development army (HDA), 50 (13⋅2 %) from community conversation (CC), 36 (9⋅9 %) from health facility (HF), and 89 (24⋅4 % from these three sources (HAD, CC, and HF). Nearly half (49⋅5 %) did not know recommended breastfeeding frequency, 86⋅2 % did not know critical hand washing times, 22⋅8 % did not know complementary food starting time, 45⋅3 % perceive that feeding a child with bottle was appropriate and 77⋅6 % did not know about minimum acceptable diet and minimum child dietary diversity. Most mothers (84⋅8 %) started complementary food feeding when their children were 6 months aged. Nearly 41 % of mothers practiced bottle-feeding. About 90 % of children were fed breast milk 24 h before survey date (Table 3).

**Table 3.** Knowledge of mothers/caregivers with children aged 6–23 months on selected infant and young child feeding practices in South-East Ethiopia (*n* = 580), February 2022

Variables	Frequency	Percent
Know age appropriate type food preparation^a^		
No	209	36⋅0
Yes	371	64⋅0
Know responsive feeding		
No	442	76⋅2
Yes	138	23⋅8
Know breastfeeding frequency		
No	293	50⋅5
Yes	287	49⋅5
Know critical hand washing times		
No	500	86⋅2
Yes	80	13⋅8
Know complementary food starting time		
No	132	22⋅8
Yes	448	77⋅2
Know importance of starting complementary feeding at 6 months		
No	174	30⋅0
Yes	406	70⋅0
Know the optimal duration of breastfeeding		
No (<24 months)	88	15⋅2
Yes (≥24 months)	492	84⋅8
Know the minimum acceptable diet		
No	450	77⋅6
Yes	130	22⋅4
Know the risk associated with nipple bottle-feeding		
No	263	45⋅3
Yes	317	54⋅7
Know the benefits of breast milk feeding to child		
No	174	30⋅0
Yes	406	70⋅0
Know the time for initiation of breastfeeding		
No	88	15⋅2
Yes (within 30 min)	492	84⋅8
Know the minimum child dietary diversity		
No	450	77⋅6
Yes	130	22⋅4
Fed breast milk day and night, yesterday		
No	55	9⋅5
Yes	525	90⋅5
At what age did you start CF		
<6 or >6 months	88	15⋅2
At 6 months	492	84⋅8
Practiced bottle-feeding		
No	338	58⋅3
Yes	242	41⋅7
Knowledge on IYCFP		
Good	365	62⋅9
Low	215	37⋅1

IYCFP,  Infant and Young Child Feeding Practice; CF, complementary feeding.

a Consistency, thickness, and variety of food prepared to start complementary feeding at 6 months and onwards.

### Dietary intake of children

**Fig. 1.**
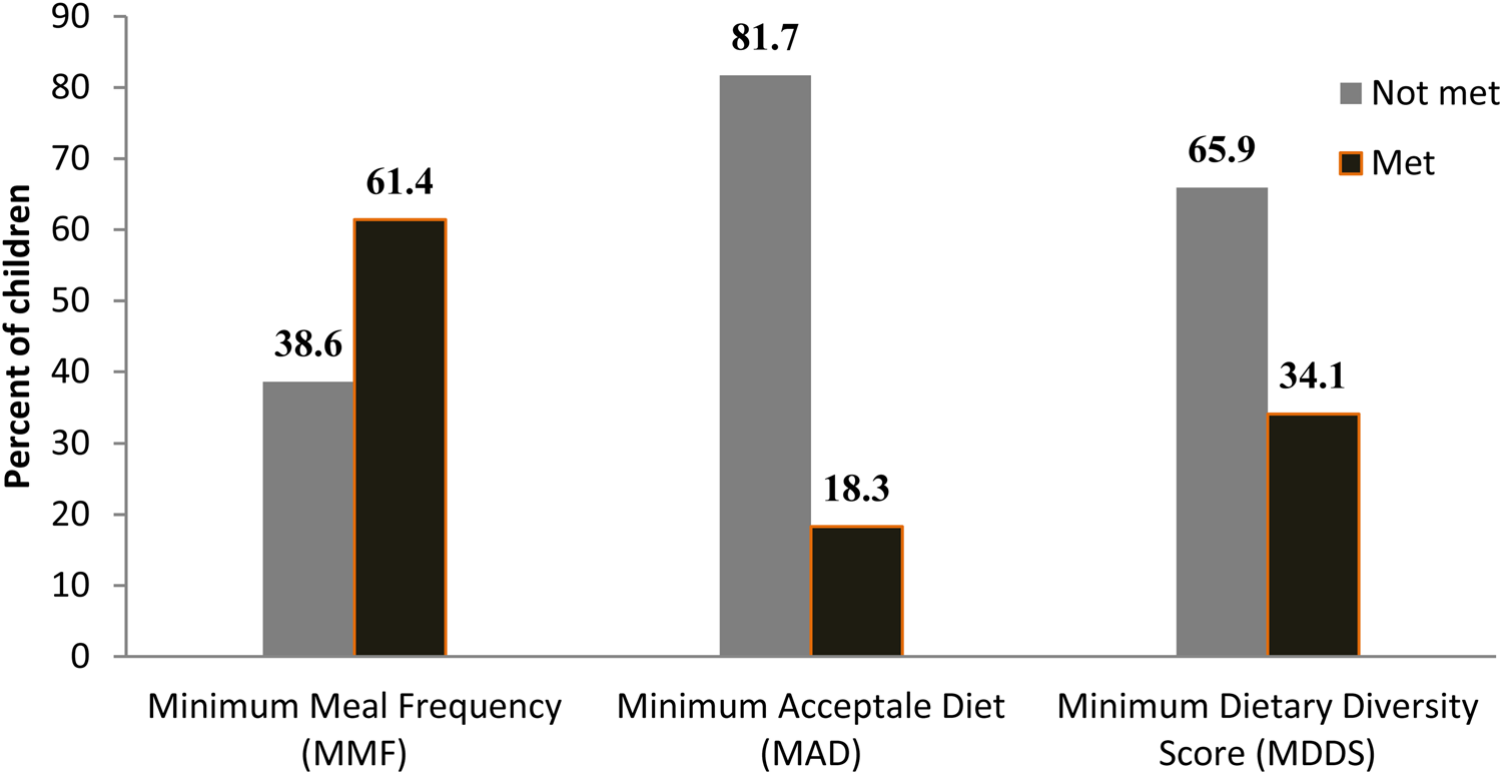
Child MMF, MAD, and MDDS status 24 h prior to the date of survey in Lemubilbilo Woreda of Oromia region, South-East Ethiopia (*n* 580), February 2022

Only 34⋅1 % of children met recommended MDDS, 61⋅4 % met MMF based on their breastfeeding status and 18⋅3 % (*n* 106) met MAD 24 h prior to the survey date (Fig. 1). Only 8⋅4 and 2⋅9 % of children ate flesh and organ meat within 24 h before the survey date. Milk and milk products, fish, and eggs were consumed by 45⋅7, 6⋅4, and 28⋅4 % of children, respectively. In addition, 38 % of children did not consume any kind of animal source foods (ASF), which did not include breast milk feeding, within 24 h prior to the date of data collection. Prevalence of ASF consumption was the least among children aged 6–8 months (6⋅7 %) compared to the 9–11 months aged (13 %) and 12–23 months aged (43⋅6 %) children (Table 4).

**Table 4.** Dietary intake of children aged 6–23 months in South-East Ethiopia (*n* = 580), 2022

Variables	6–8	9–11	12–23	Total (6–23)
Fed breast milk day and night, yesterday				
No	0 (0⋅0)	4 (0⋅7)	51 (8⋅8)	55 (9⋅5)
Yes	71 (12⋅2)	122 (21⋅0)	332 (57⋅2)	525 (90⋅5)
Porridge made from CRT				
No	15 (2⋅6)	14 (2⋅4)	45 (7⋅8)	74 (22⋅8)
Yes	56 (9⋅7)	112 (19⋅3)	327 (56⋅4)	506 (87⋅2)
Food made from pulses/legumes and Nuts				
No	45 (7⋅8)	73 (12⋅6)	211 (36⋅4)	334 (57⋅6)
Yes	26 (4⋅5)	53 (9⋅1)	161 (27⋅8)	246 (42⋅4)
Yellow pumpkin, squash or OFSP in any form^a^				
No	57 (9⋅8)	94 (16⋅2)	297 (51⋅2)	448 (77⋅2)
Yes	14 (2⋅4)	32 (5⋅5)	86 (14⋅8)	132 (22⋅8)
Dark green leafy fruits and vegetables				
No	59 (10⋅2)	98 (16⋅9)	294 (50⋅7)	451 (77⋅8)
Yes	12 (2⋅1)	28 (4⋅8)	89 (15⋅3)	129 (22⋅2)
Ripe papaya, mango and other yellow fruits^a^				
No	64 (11⋅0)	107 (18⋅4)	326 (56⋅2)	497 (85⋅7)
Yes	7 (1⋅2)	19 (3⋅3)	57 (9⋅8)	83 (14⋅3)
Other fruits and vegetables				
No	66 (11⋅4)	118 (20⋅3)	336 (57⋅9)	520 (89⋅7)
Yes	5 (0⋅9)	8 (1⋅4)	47 (8⋅1)	60 (10⋅3)
Organ meat (hear, kidney, liver)				
No	71(12⋅2)	124 (21⋅4)	368 (63⋅4)	563 (97⋅1)
Yes	0 (0⋅0)	2 (0⋅3)	15 (2⋅6)	17 (2⋅9)
Meat (beef, chicken, goat, sheep or other sources^b^				
No	69 (11⋅9)	120 (20⋅7)	342 (59⋅0)	531 (91⋅6)
Yes	2 (0⋅3)	6 (1⋅0)	41 (7⋅1)	49 (8⋅4)
Fish				
No	68 (11⋅7)	119 (20⋅5)	356 (61⋅4)	543 (93⋅6)
Yes	4 (0⋅7)	7 (1⋅2)	26 (4⋅5)	37 (6⋅4)
Egg				
No	55 (9⋅5)	90 (15⋅5)	270 (46⋅6)	415 (71⋅6)
Yes	16 (2⋅8)	36 (6⋅2)	113 (19⋅5)	165 (28⋅4)
Milk and milk products (yoghurt, cheese, etc.)				
No	48 (8⋅3)	76 (13⋅1)	191 (32⋅9)	315 (54⋅3)
Yes	23 (4⋅0)	50 (8⋅6)	191 (32⋅9)	265 (45⋅7)
Meal frequency				
1 meal	18 (3⋅1)	11 (1⋅9)	10 (1⋅7)	39 (6⋅7)
2 meals	14 (2⋅4)	43 (7⋅4)	126 (21⋅7)	183 (31⋅6)
3 meals	12 (2⋅1)	33 (5⋅7)	118 (20⋅3)	163 (27⋅1)
≥4 meals	27 (4⋅7)	39 (6⋅7)	129 (22⋅2)	194 (34⋅6)
Animal source food (ASF) consumption				
Never	32 (5⋅5)	51 (8⋅8)	130 (22⋅4)	213 (36⋅7)
At least one	39 (6⋅7)	75 (13⋅0)	253 (43⋅6)	367 (63⋅3)
MMF based on breastfeeding status				
Not met	18 (3⋅1)	51 (8⋅8)	155(26⋅7)	224 (38⋅6)
Met	53 (9⋅1)	75 (13⋅0)	228 (39⋅3)	356 (61⋅4)
Dietary diversity score^c^				
≥5	21 (3⋅6)	43 (7⋅4)	134 (23⋅1)	198 (34⋅1)
<5	50 (8⋅6)	83(14⋅3)	249 (43⋅0)	382 (65⋅9)
Minimum acceptable diet				
Met	13 (2⋅2)	21 (3⋅6)	72 (12⋅4)	106 (18⋅3)
Not met	58 (10⋅0)	105 (18⋅1)	311 (53⋅6)	474 (81⋅7)

CRT,  cereals, roots, and tubers; MMF, minimum meal frequency; OFSP,  orange fleshed sweet potato.

a These food groups merged into vitamin A rich fruits and vegetables.

b These food groups merged into meat for the dietary diversity score computation.

c DDS computation included breast milk feeding.

### Prevalence of undernutrition

**Fig. 2.**
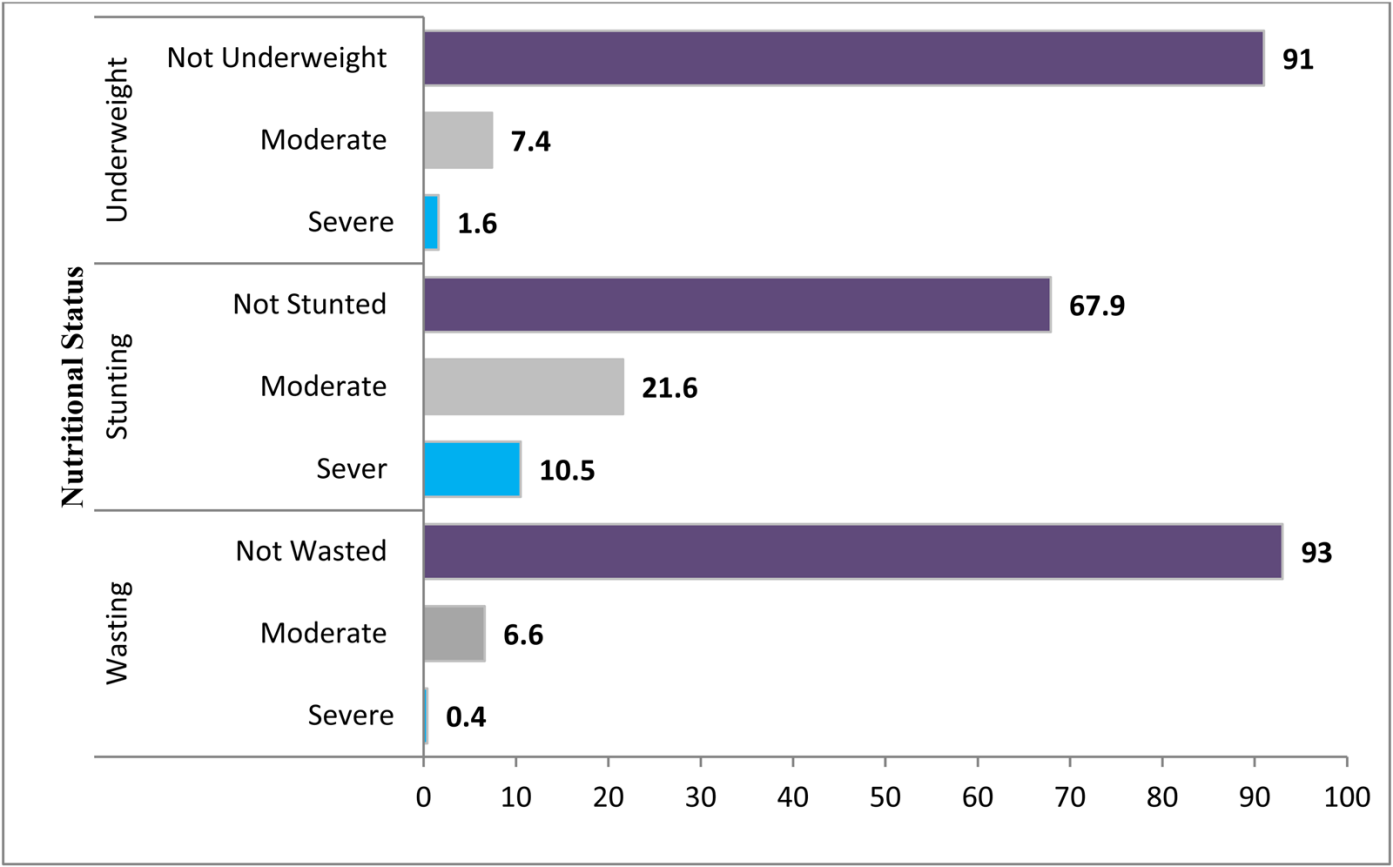
Undernutrition status on the level of severity in Lemubilbilo Woreda of Oromia region, South-East Ethiopia (*n* 580), February 2022.

**Fig. 3.**
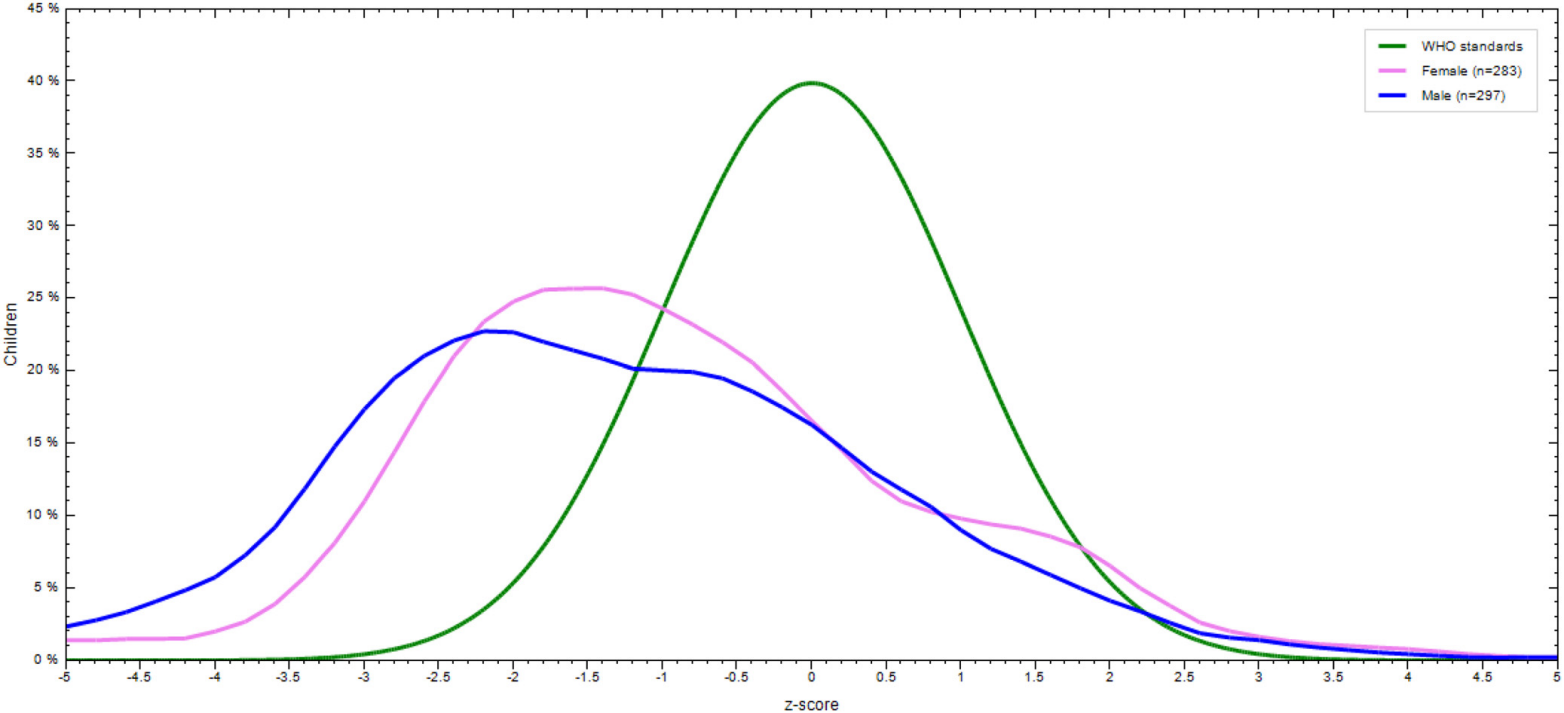
Height/length-for-Age (HAZ) WHO anthro-growth curve of 6–23 months old children by sex in Lemubilbilo Woreda of Oromia region, South-East Ethiopia, February 2022.

**Fig. 4.**
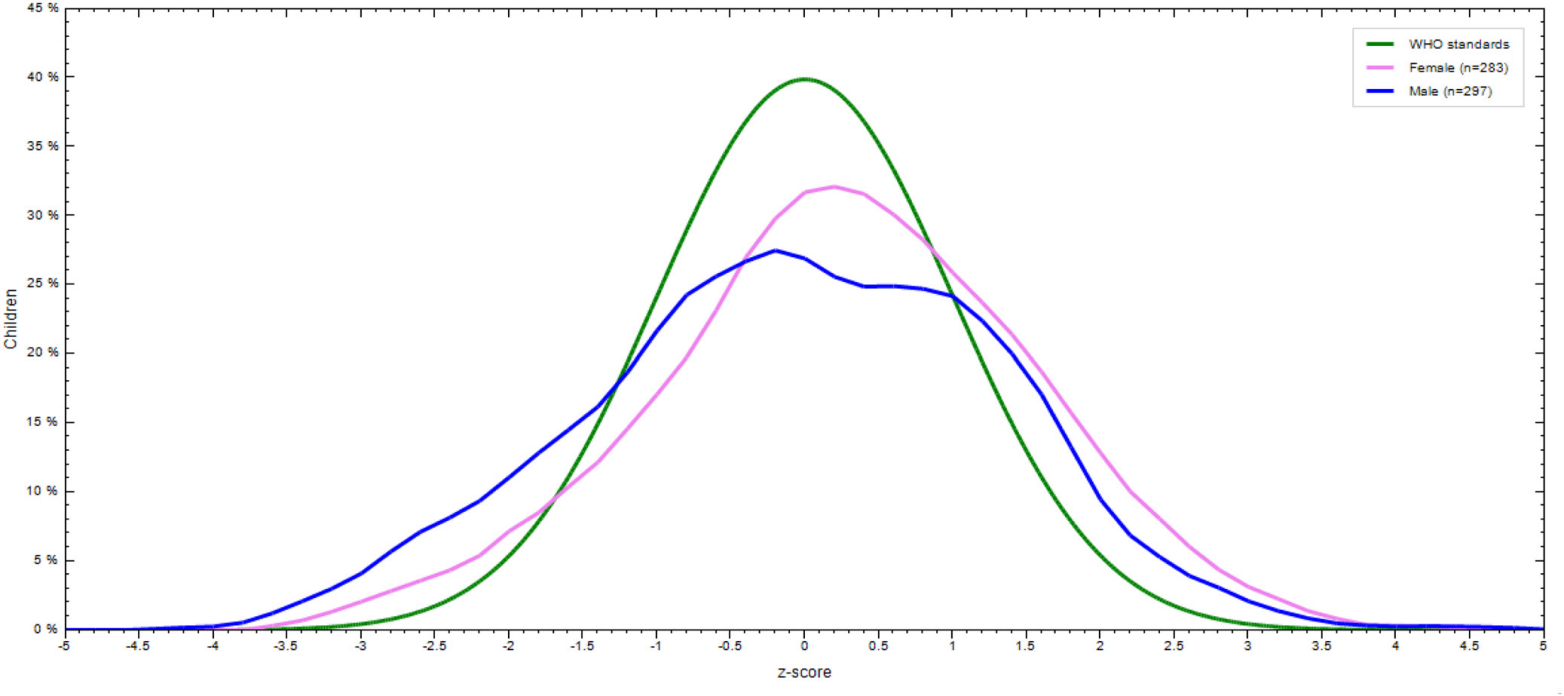
Weight-for-height/length (WHZ) WHO anthro curve of 6–23 months old children by sex in Lemubilbilo Woreda of Oromia region, South-East Ethiopia, February 2022.

**Fig. 5.**
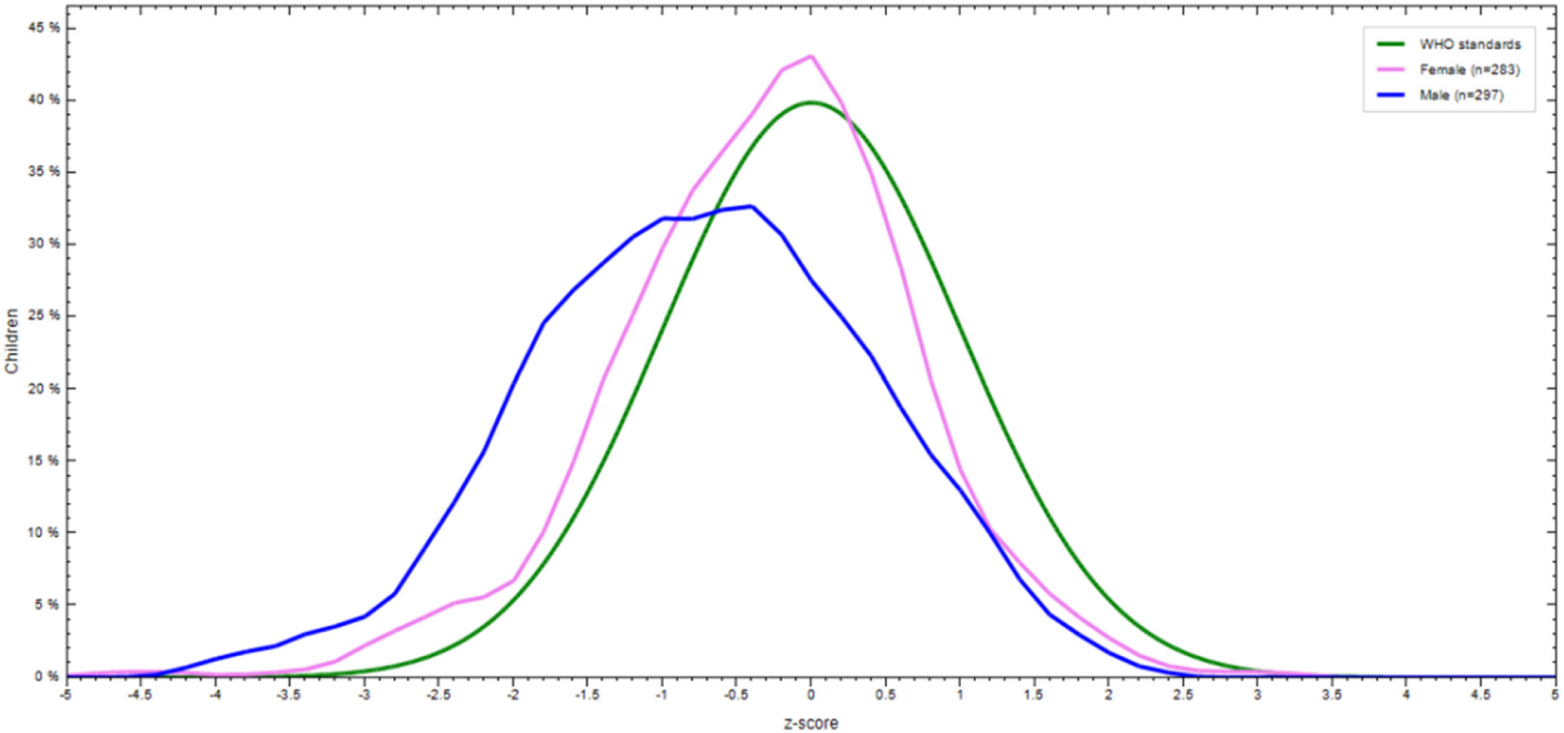
Weight-for-Age (WAZ) WHO anthro curve of 6–23 months old children by sex in Lemubilbilo Woreda of Oromia region, South-East Ethiopia, February 2022.

The prevalence of stunting, wasting, and underweight were 32⋅1 % (95 % CI: 28⋅3 %, 35⋅9 %), 7 % (95 % CI: 4⋅9 %, 9⋅1 %), and 9 % (95 % CI: 6⋅7 %, 11⋅3 %), respectively. The magnitude of all three forms of undernutrition is higher in 12–23 months aged children (Table 5). Severe wasting, stunting, and underweight were 0⋅4, 10⋅5, and 1⋅6 %, respectively (Fig. 2). The sex-specific WHO standard growth curve for Height/length-for-Age (HAZ), Weight-for-Height/length (WHZ), and Weight-for-Age (WAZ) is indicated in Figs. 3–5, respectively.

**Table 5.** Prevalence of undernutrition among children aged 6–23 months in South-East Ethiopia (*n* = 580), February 2022

Nutritional status	Age of children in months, *n* (%)		
	6–11	12–23	Total (6–23)
Waste			
Yes	11 (5⋅6)	29 (7⋅6)	40 (7⋅0)
No	186 (94⋅4)	354 (92⋅4)	540 (93⋅0)
Stunted			
Yes	62 (31⋅5)	124 (32⋅4)	186 (32⋅1)
No	135 (68⋅5)	259 (67⋅6)	394 (67⋅9)
Underweighted			
Yes	22 (11⋅2)	30 (7⋅8)	52 (9⋅0)
No	175 (89⋅8)	353 (92⋅2)	528 (90⋅0)

### Factors associated with undernutrition

Factors which were significantly associated with higher risk of children growth stunting are: being male (AOR = 1⋅75), not using GMP service (AOR = 1⋅50), household food insecurity (AOR = 1⋅67), lack of access to improved water (AOR = 2⋅26), and bottle-feeding (AOR = 1⋅64). On the other hand, wasting was significantly predicted by being male (AOR = 3⋅02), low knowledge of mothers about recommended IYCFP (AOR = 3⋅89), not listening to the radio/television (AOR = 3⋅69), having a history of fever 2 weeks before survey date (AOR = 3⋅39), bottle feeding (AOR = 3⋅58) and household food insecurity (AOR = 3⋅77). In addition, childhood underweight was significantly predicted by being male (AOR = 3⋅44), not using GMP service (AOR = 2⋅00), having history of fever (AOR = 4⋅24), lack of knowledge about optimal duration of breastfeeding (AOR = 3⋅58), having low knowledge of caregivers about IYCFP (AOR = 2⋅21), household food insecurity (AOR = 2⋅04) and lack of access to improved water (AOR = 3⋅00) (Table 6).

**Table 6.** Factors associated with undernutrition among children aged 6–23 months in South-East Ethiopia (*n* = 580), 2022

		Stunting				
Variables		Yes	No	COR (95 %CI)	AOR (95 %CI)	*P*-value
Sex of the child	Female	73	211	1	1	
	Male	113	183	1⋅78 (1⋅25, 2⋅54)	1⋅75 (1⋅21, 2⋅54)	0⋅003
GMP service utilisation	Yes	97	255	1	1	
	No	89	139	1⋅68 (1⋅18, 2⋅39)	1⋅50 (1⋅14, 2⋅18)	0⋅001
Food security	Food secure	75	195	1	1	
	Food Insecure	111	199	1⋅15 (1⋅02, 2⋅06)	1⋅67 (1⋅14, 2⋅42)	0⋅008
Paternal education	Formal education	156	300	1	1	
	No formal education	30	94	1⋅63 (1⋅04, 2⋅57)	1⋅76 (1⋅08, 2⋅83)	0⋅021
Source of water	Improved	101	130	1	1	
	Not improved	85	264	2⋅41 (1⋅69, 2⋅44)	2⋅26 (1⋅55, 3⋅29)	<0⋅001
Bottle-fed	No	66	197	1	1	
	Yes	120	197	1⋅82 (1⋅26, 2⋅60)	1⋅64 (1⋅12, 2⋅39)	0⋅011
		Wasting				
		Yes	No			
Sex of the child	Female			1	1	
	Male			2⋅69 (1⋅32, 5⋅50)	3⋅02 (1⋅34, 6⋅84)	0⋅008
Knowledge of IYCFP	Yes	13	188	1	1	
	No	27	352	3⋅89 (1⋅96, 7⋅71)	3⋅84 (1⋅79, 8⋅25)	0⋅001
Fever	No	27	473	1	1	
	Yes	13	67	3⋅39 (1⋅67, 6⋅91)	3⋅25 (1⋅42, 7⋅42)	0⋅005
Listen radio/watch TV	Yes	16	384	1	1	
	No	24	156	3⋅69 (1⋅91, 7⋅13)	2⋅33 (1⋅12, 4⋅86)	0⋅024
Currently BF	Yes	29	496	1	1	
	No	11	44	4⋅27 (2⋅00, 9⋅13)	4⋅39 (1⋅80, 10⋅68)	0⋅002
Bottle-fed	Yes	32	285	1	1	
	No	8	255	3⋅58 (1⋅62, 7⋅91)	3⋅43 (1⋅43, 8⋅24)	0⋅006
Food security	Food secure	8	262	1	1	
	Insecure	32	278	3⋅77 (1⋅71, 8⋅33)	3⋅42 (1⋅47, 7⋅76)	0⋅004
		Underweight				
		Yes	No			
Sex of child	Female	14	270	1	1	
	Male	38	258	2⋅84 (1⋅50, 5⋅36)	3⋅44 (1⋅71, 6⋅93)	0⋅01
GMP service utilisation	Yes	21	331	1	1	
	No	31	197	2⋅48 (1⋅38, 4⋅43)	2⋅00 (1⋅10, 3⋅72)	0⋅009
Fever in last 2 weeks	No	32	468	1	1	
	Yes	60	20	4⋅87 (2⋅62, 9⋅06)	4⋅24 (2⋅14, 8⋅37)	0⋅001
Knew optimal duration of BF	Yes	34	458	1	1	
	No	18	70	3⋅46 (1⋅85, 6⋅45)	3⋅58 (1⋅75, 7⋅31)	0⋅001
Knew IYCFP	Yes	23	342	1	1	
	No	29	186	2⋅32 (1⋅30, 4⋅12)	2⋅21 (1⋅20, 4⋅05)	0⋅011
Food security status	Food secure	17	253	1	1	
	Food insecure	35	275	1⋅89 (1⋅04, 346)	2⋅04(1⋅04, 3⋅98)	0⋅037
Source of water	Yes	32	199	1	1	
	No	20	329	2⋅64 (1⋅47, 4⋅75)	3⋅00 (1⋅53, 5⋅86)	0⋅001

BF, breastfeeding; IYCFP, infant and young child feeding practice; GMP, growth monitoring and promotion; TV, television.

## Discussion

The prevalence of stunting, wasting and underweight in this study is lower compared to the national food and nutrition strategy (FNS) baseline survey preliminary report in which stunting, wasting and underweight were 39, 11, and 21 % respectively.^(25)^ The result of this study is also lower than a meta-analysis reported in Ethiopia where 42, 33, and 15 % of 6–23 months aged children were stunted, wasted, and underweighted respectively.^(26)^ Similarly, a higher prevalence of stunting (39 %) was reported by other studies at the African level.^(27)^ This variation by the prevalence of stunting and underweight compared to FNS baseline could be because the FNS survey was a country-wide study conducted with a large sample size and a socio-culturally diverse population. It also might be due to the difference in the seasonality of data collection as this study was conducted during the crop harvesting period. Another possible explanation could be due to the presence of different Non-Government Organisations (NGOs) that work on IYCFP feeding practices, nutrition education and health promotion services.

In this study, the risk of undernutrition was higher in males than females. This finding is consistent with a meta-analysis study conducted to examine the sex differences in undernutrition.^(28,29)^ Other studies also showed that males were more likely to be stunted,^(28,30–32)^ wasted,^(28,33)^ and underweight^(29,34)^ than females. There is an exception in studies where the risk is the reverse and females are more likely to be undernourished than males.^(35)^ This could be because different societies have different cultures and give different positions to males and females, which could affect fair childcare practices. Another possible explanation could be due to maternal nutrition during pregnancy, childbirth weight, and resilience to environmental stress could drive variation in undernutrition by sex. This is supported by scientific evidence that male vulnerability in response to environmental stress in early life has seen infants manifesting greater morbidity.^(36)^ Supportive findings have been reported by another scholar in which males were more valuable to environmental influence.^(37)^ In our context, a difference between male and female children and risk of undernutrition needs further research.

The risk of being stunted and underweight is lower among children who utilised recommended age appropriate GMP service compared to those who were not utilised. Consistent result was demonstrated the association between stunting and not using GMP service in other similar studies.^(30)^ This could be because the primary focus of GMP is to provide opportunities for health workers to assess a child's health and nutritional status and offer counselling on proper feeding, influence caregivers to take action, and affect family-level decision-making on child feeding. With these regards, GMP-followed mothers acquire more knowledge on proper child feeding and care practices, and are psychosocially stimulated on good child nutrition and health. On the contrary, other health services (ANC and PNC) utilisation did not show significant association with all three forms of undernutrition. This might be due to relative difference in sample size and also variation in frequency of ANC and PNC visits among mothers in different studies. The result is not conclusive and doesn't mean that utilisation of these services has no effect on child nutritional status. Further research is needed on the effect of these services including quality of services on child undernutrition.

In this study, children from food-insecure households were more likely to be stunted, wasted, and underweight as compared to food-secure groups. The strong association between household food insecurity and child growth stunting,^(31,38,39)^ underweight,^(40,41)^ and wasting^(39)^ was demonstrated by other studies. This could be because food insecurity is a more proximal factor of inadequate dietary intake^(42–44)^ and disease resulting from inadequate nutrient intake.^(44)^ This finding implies the potential impact of household food insecurity on both chronic and acute child undernutrition. MMF, DDS, and MAD did not show a statistically significant association with all three forms of undernutrition. This could be because DDS, MMF, and MAD were computed based on one 24 h dietary recall which may not be adequate to reflect usual true intake. This does not mean that these practices are not important for child growth and health, but the longitudinal study may explore more accurate associations.

This study demonstrated a positive association of lack of paternal education with stunting. Supporting results were demonstrated in other similar studies.^(45–49)^ This could be because educated fathers earn more household income,^(50)^ be more knowledgeable about healthy diets, and have a higher influence on household decision-making, maximising improved child-care practices compared to uneducated parents. In contrast to other similar studies,^(34,35,39,51)^ maternal education did not show a significant association with all three forms of undernutrition in this study. This could be explained by the true association that might be masked by seasonal food availability. The result is not conclusive but needs pooled analysis to explore a more precise result.

The risk of stunting and being underweight was higher in children from households of an unimproved source of water category. Similar findings were reflected in other studies where the unimproved water source positively predicted stunting^(30,47)^ and underweight.^(47)^ This could be because using unimproved water can cause intestinal parasitic and other infections like diarrhoea leading to decreased nutrient absorption,^(52)^ long-term nutritional deficiency, and growth retardation.

A positive association of fever with wasting and being underweight was observed in this study. Similar supportive evidence has witnessed the impact of frequent fever on a child's physical growth^(53)^ and underweight.^(54)^ This is because infection reduces dietary intake,^(44)^ through loss of appetite and elevated nutrient demand due to fever affecting linear growth,^(55)^ and acute body fat and protein storage.

WHO (2021) recommends continuous breastfeeding for up to 2 years and discourages bottle-feeding for healthy growth and nutrition^(56)^ In this study, bottle-fed children were more likely to be stunted and wasted. Similar result was demonstrated in other studies where stunting,^(30,56–59)^ and acute undernutrition is strongly predicted by bottle-feeding.^(34)^ Bottle-fed children could be exposed to pathogens which result in recurrent childhood illness and undernutrition. Children who had been breastfed during the data collection period were less likely to be wasted as compared to non-breast milk-fed children. This might be due to children's continued breast milk feeding that could meet a substantial portion of their energy and nutrient needs in their diet. It is vital during illness, while sick children often have little appetite for solid food and can help prevent dehydration.^(56)^

Maternal knowledge of appropriate infant and young child feeding practice is an immediate factor for dietary intake and illness prevention.^(44)^ In this study, caregivers with good knowledge of IYCFPs were less likely to be wasted and underweight. This is because caregivers with good knowledge have a high probability to follow recommended feeding and caring practices. Furthermore, not listening to the radio/watching television is positively associated with acute undernutrition. This could be because mothers/caregivers who were exposed to television/radio health and nutrition messages can acquire extra IYCF knowledge and skills, and practice healthy childcare as currently the importance of taking vitamin supplementation capsule, child vaccination, appropriate breastfeeding and complementary feeding messages, hygienic practice and maternal nutrition has delivering as key message through these media.

This finding gives some hints for researchers, government sectors, stakeholders and mothers/caregivers on child nutrition. Hence, investigations should continue to focus on analysing the cause and effect relationship of nutrition situation in the area using longitudinal study, and considering quality of health services and seasonality. Government sectors, stakeholders working on child nutrition, and mothers/caregivers should focus on preventive measures of childhood undernutrition.

### Strength and limitations

This study is the first small-scale survey in the study area and included a large sample size. One of the limitations of the study is that fact that only one-day dietary recall was collected which might not reflect the true usual dietary intake. There might be professional bias, and recall bias about the dietary intake and feeding practices. There might also be potential social desirability bias on wealth and food security responses. Finally, preconception maternal nutrition, childbirth weight, access to and quality of health and nutrition related services are not assessed. Since the design of the research is cross sectional, we recommend further study about the nature of cause and effect relations between undernutrition and associated factors.

## Conclusion

The prevalence of stunting is alarmingly common while wasting and underweight are sub-optimal among children aged 6–23 months. Being male sex and household food insecurity were commonly significantly associated with all three forms of undernutrition whereas paternal education, not listening to the radio/watching television and lack of knowledge on optimal duration of breast feeding were independently associated with stunting, wasting and underweight respectively. Factors commonly associated with: stunting and underweight were low GMP service utilisation and lack of access to clean water; wasting and underweight were lack of knowledge on optimal duration of breast feeding and history of fever 2 weeks before survey; and stunting and wasting was bottle feeding practice. Based on this finding, addressing associated factors of undernutrition in the study area are crucial for improving the young children nutrition, and these include taking greater measures to prevent infectious disease, providing basic education especially for fathers, enhancing media access, ensuring household food security, enhancing women knowledge about IYCFP, enhancing access to improved water, promoting GMP service utilisation, extended breastfeeding, and discouraging bottlefeeding practices.
